# Fine-tuning of noise in gene expression with nucleosome remodeling

**DOI:** 10.1063/1.5021183

**Published:** 2018-05-07

**Authors:** Melina R. Megaridis, Yiyang Lu, Erin N. Tevonian, Kendall M. Junger, Jennifer M. Moy, Kathrin Bohn-Wippert, Roy D. Dar

**Affiliations:** 1 Department of Bioengineering, University of Illinois at Urbana-Champaign, Urbana, Illinois 61801, USA; 2 Carl R. Woese Institute for Genomic Biology, University of Illinois at Urbana-Champaign, Urbana, Illinois 61801, USA; 3 Center for Biophysics and Quantitative Biology, University of Illinois at Urbana-Champaign, Urbana, Illinois 61801, USA

## Abstract

Engineering stochastic fluctuations of gene expression (or “noise”) is integral to precisely bias cellular-fate decisions and statistical phenotypes in both single-cell and multi-cellular systems. Epigenetic regulation has been shown to constitute a large source of noise, and thus, engineering stochasticity is deeply intertwined with epigenetics. Here, utilizing chromatin remodeling, we report that Caffeic acid phenethyl ester (CA) and Pyrimethamine (PYR), two inhibitors of BAF250a, a subunit of the Brahma-associated factor (BAF) nucleosome remodeling complex, enable differential and tunable control of noise in transcription and translation from the human immunodeficiency virus long terminal repeat promoter in a dose and time-dependent manner. CA conserves noise levels while increasing mean abundance, resulting in direct tuning of the transcriptional burst size, while PYR strictly increases transcriptional initiation frequency while conserving a constant transcriptional burst size. Time-dependent treatment with CA reveals non-continuous tuning with noise oscillating at a constant mean abundance at early time points and the burst size increasing for treatments after 5 h. Treatments combining CA and Protein Kinase C agonists result in an even larger increase of abundance while conserving noise levels with a highly non-linear increase in variance of up to 63× untreated controls. Finally, drug combinations provide non-antagonistic combinatorial tuning of gene expression noise and map a noise phase space for future applications with viral and synthetic gene vectors. Active remodeling of nucleosomes and BAF-mediated control of gene expression noise expand a toolbox for the future design and engineering of stochasticity in living systems.

## INTRODUCTION

Our ability to control and engineer intracellular biological processes is hampered by stochastic gene expression (or “noise”) resulting from the dynamic and heterogeneous cell environment. Heterogeneity resulting from multiple stochastic processes including intracellular gene expression, cell to cell signaling, and environmental factors propagates up to populations of cells, tumors, and tissue patterning.^1–3^ Future bioengineering of living systems will require the establishment of fundamentals and tools to cope with, exploit, design, and engineer stochasticity.

To date, noise in gene expression has been studied for its sources^4^ and consequences in the decision-making of diverse organisms across all kingdoms of life.^2^ Most recently, these fundamentals have been modeled and applied towards the active manipulation and control of noise for biasing stochastically driven systems into a desired state. These include synthetic gene circuits with multiple inputs,^5,6^ fitness levels of yeast challenged by fluctuating environments,^7^ competence state in *Bacillus subtilis*,^8^ commitment of yeast to the phosphate starvation program,^9^ modifying ribosomal binding sites to control *Escherichia coli* noise,^10^ cell-free gene expression systems with controlled reaction volumes,^11^ epigenetic states of embryonic stem cells,^12,13^ and exogenous control of human immunodeficiency virus (HIV) gene expression with noise modulating compounds.^14,15^ This study investigates tuning gene expression noise of a promoter by dose- and time-dependent treatment of multiple drugs that target the Brahma-associated factor (BAF) nucleosome remodeling complex.

Promoter nucleosome occupancy has been linked to stochastic gene expression in eukaryotes.^16^ Members of the SWI/SNF (SWItching/Sucrose Non-Fermenting) nucleosome remodeling complex family such as BAF and Polybromo-associated BAF (PBAF) are integral to chromatin remodeling and transcriptional regulation of development and pluripotency.^17^ They are also involved in HIV^18,19^ and are found to be heavily enriched with mutations in cancer.^20^ Investigations reveal that BAF nucleosome positioning of a proviral nucleosome (nuc-1) downstream of the transcriptional start site of the HIV long terminal repeat (LTR) promoter represses transcription.^18^ Silencing RNA targeting of specific BAF subunits leads to positional relaxation of nuc-1 and reactivation of latent HIV.^18^ In addition, investigations of the HIV LTR show that common chromatin modifiers, noise enhancement, and synergistic reactivation of the latent viral state are closely related.^14,15,21^

In a recent study, BAF inhibitors (BAFis), Pyrimethamine (PYR) and Caffeic acid phenethyl ester (CA or CAPE), were shown to synergize reactivation of latent HIV with transcriptional activators.^22^ Consistent with previous studies of HIV drug synergies, this suggests that BAF inhibitors may provide a novel drug class to enhance and finely tune transcriptional noise of the LTR promoter.^14,18,23^ Here, we investigate if BAF inhibition and modification of nucleosome occupancy patterning provide a mechanism for tunable control of gene expression fluctuations generated from the HIV promoter. We find that BAFis can independently modulate the transcriptional initiation rate (burst frequency) and the transcriptional burst size. Combining CA with a class of transcriptional activators, Protein Kinase C agonists (PKCas), like tumor necrosis factor (TNF) alpha, Prostratin, or Phorbol 12-myristate 13-acetate (PMA), we observe an additional increase in the translational burst size. When combined with PYR, a three-drug cocktail simultaneously modulates the transcriptional burst size and frequency along with the translational burst size. Noise modulation is demonstrated with a fold-change increase of ∼45–63× in variance and ∼1.4× in transcriptional burst frequency in a dose dependent manner.

## RESULTS

### The 2-state model for episodic transcription

**FIG. 1.**
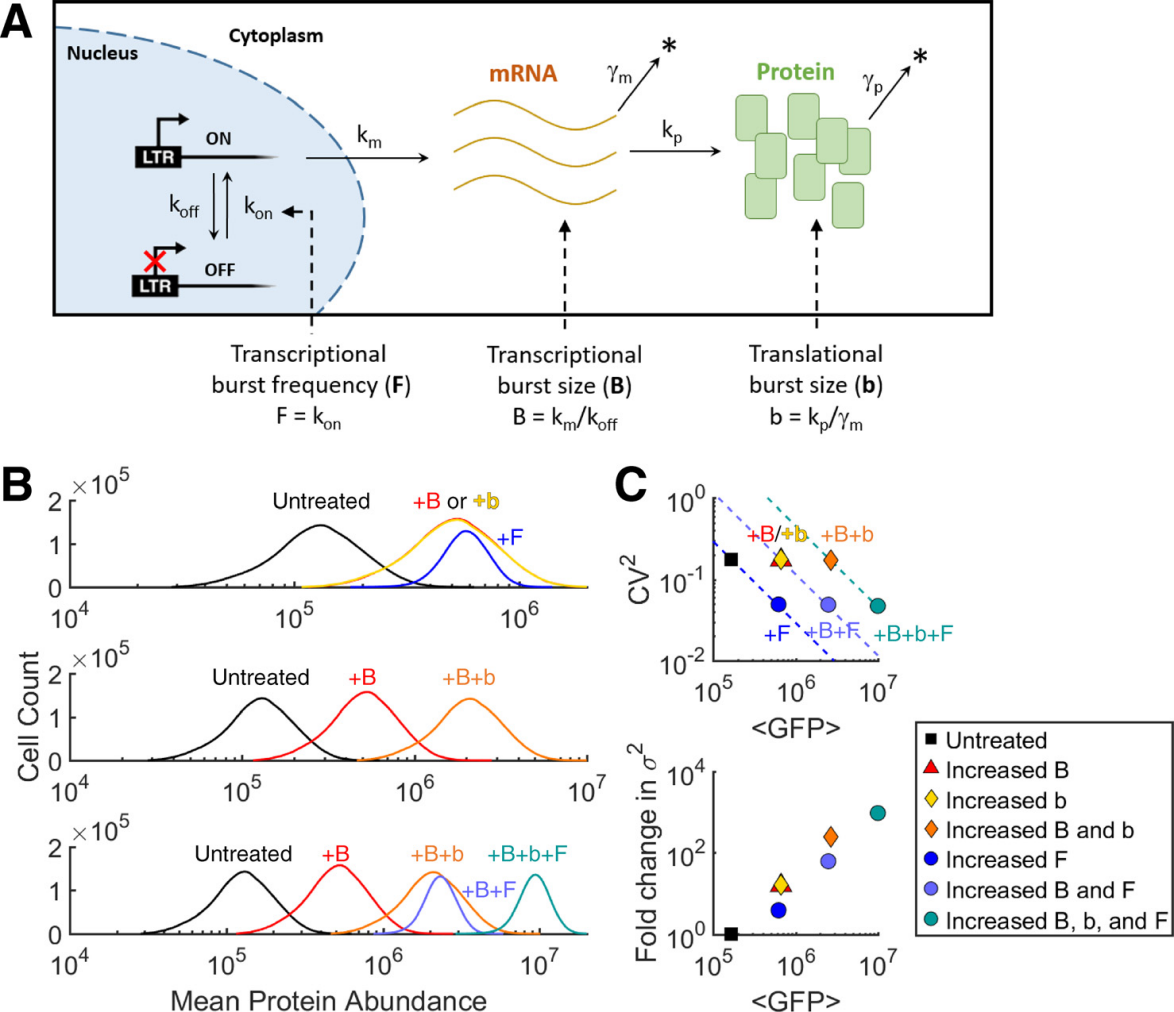
One- and two-input gene expression noise generation by modulating transcription and translation. (a) Illustration of the 2-state model of a slow switching gene promoter between the “on” state (with active transcription) and the “off” state (without transcription). The effect of changing the mRNA burst size “B” (No. of mRNA produced per “on” state cycle), the protein burst size “b” (No. of proteins produced per mRNA lifetime), and/or the burst frequency “F” (rate of promoter initiation into the “on” state) of the cell population. (b) Stochastic simulations for the gene circuit in panel (a) using the Gillespie algorithm^53^ and parameters (supplementary material and Ref. 23). Distributions of cells according to their protein abundance under different conditions and changes in B, b, and/or F are plotted. (c) Change in the noise magnitude (measured by the coefficient of variation squared, CV^2^) versus mean protein abundance (⟨P⟩) and fold change in variance versus mean protein abundance, under the same labeled conditions across each row in (b).

The LTR promoter has been investigated for its episodic or burst noise and role in viral decision-making between latent and active production states.^14,24^ Episodic transcription of the promoter has been described quantitatively by a simplified 2-state model of promoter activity^23,25–27^ [Fig. 1(a)]. The 2-state model consists of the promoter in an OFF state, with RNA polymerase II stalled behind a nucleosome stabilized by the BAF remodeling complex, and an ON state, initiated at a rate k_on_, in which multiple pol II are released to transcribe before the promoter decays back to the inactive state at a rate k_off_. k_on_ is also known as the burst frequency (F). Transcription occurs in the ON state at a rate k_m_, and the number of mRNA produced per activity pulse of the promoter (T_on_ = 1/k_off_) is defined as the transcriptional burst size (B = k_m_*T_on_ = k_m_/k_off_). Translation occurs at a rate k_p_, and the burst of proteins translated per mRNA lifetime is defined as the translational burst size (b = k_p_/γ_m_), with γ_m_ being the decay rate for messenger RNA (mRNA). For this model, the following equations for the expression noise magnitude, quantified by the coefficient of variation squared (CV^2^ = σ^2^/⟨P⟩^2^), show that modulation of noise and mean protein abundance can occur by changing B, F, and/or b (Fig. 1)^9,23,25,26,28–30^

$$\left\langle P\right\rangle =\frac{bBF}{\gamma_{P}},$$ (1)

$${CV}^{2}=\frac{\gamma_{P}(b+1)B}{BbF}\approx \frac{\gamma_{P}}{F}.$$ (2)

Here, the promoter is assumed to be at low activity levels where (i) k_off_ ≫ k_on_, (ii) k_off_ ≫ k_m_, (iii) k_off_ ≫ γ_p_, (iv) k_m_ ≫ (γ_m_ + γ_p_), and (v) the approximation in (2) holds for b ≫ 1.

According to these expressions, the increase in abundance while conserving a constant noise level would indicate the increase in the burst size of either transcription (B) or translation (b), increased variance, and constant burst frequency (F).^11,29^ The increase in protein abundance with a constant burst size (B and b) would indicate the increase in burst frequency (F) and constant variance, with noise inversely proportional to mean protein abundance.^23,26,28^

Noise modulation at levels of transcription and translation requires multiple signaling inputs to control each noise source.^6^ Stochastic simulations demonstrate how the LTR promoter can increase expression from an untreated state to the same mean abundance with different levels of noise by changing B, F, or b [Figs. 1(b) and 1(c) and Eq. (1)]. In addition, simultaneously modulating either two or all three noise “dials” can hypothetically cover a large range of noise phase space (e.g., B + b, B + F, or B + b + F) [Fig. 1(c)].^6,23,31^

A recent drug screen detected a subset of small molecule compounds that target the BAF nucleosome remodeling complex.^32^ Two of the leading compounds, PYR and CA, shown to degrade the BAF250a subunit,^22^ were further characterized to target the HIV promoter and activate latent HIV-1 in Jurkat T-cell lines.^22^ In addition, another recent study demonstrates that BRD4 promotes HIV-1 latency by binding the BAF250a and BRG1 subunits.^33^ Consistent with this study, Rafati *et al.* demonstrate nucleosome relaxation, LTR promoter activation, and latent reactivation of full-length HIV by silencing with siBAF250a.^18^ Collectively, these studies motivate the hypothesis that *exogenous drug treatments inhibiting BAF nucleosome remodeling can be used to precisely and finely tune the noise of LTR gene expression.*

### BAFi compounds can orthogonally tune the burst size and frequency in a dose responsive manner

**FIG. 2.**
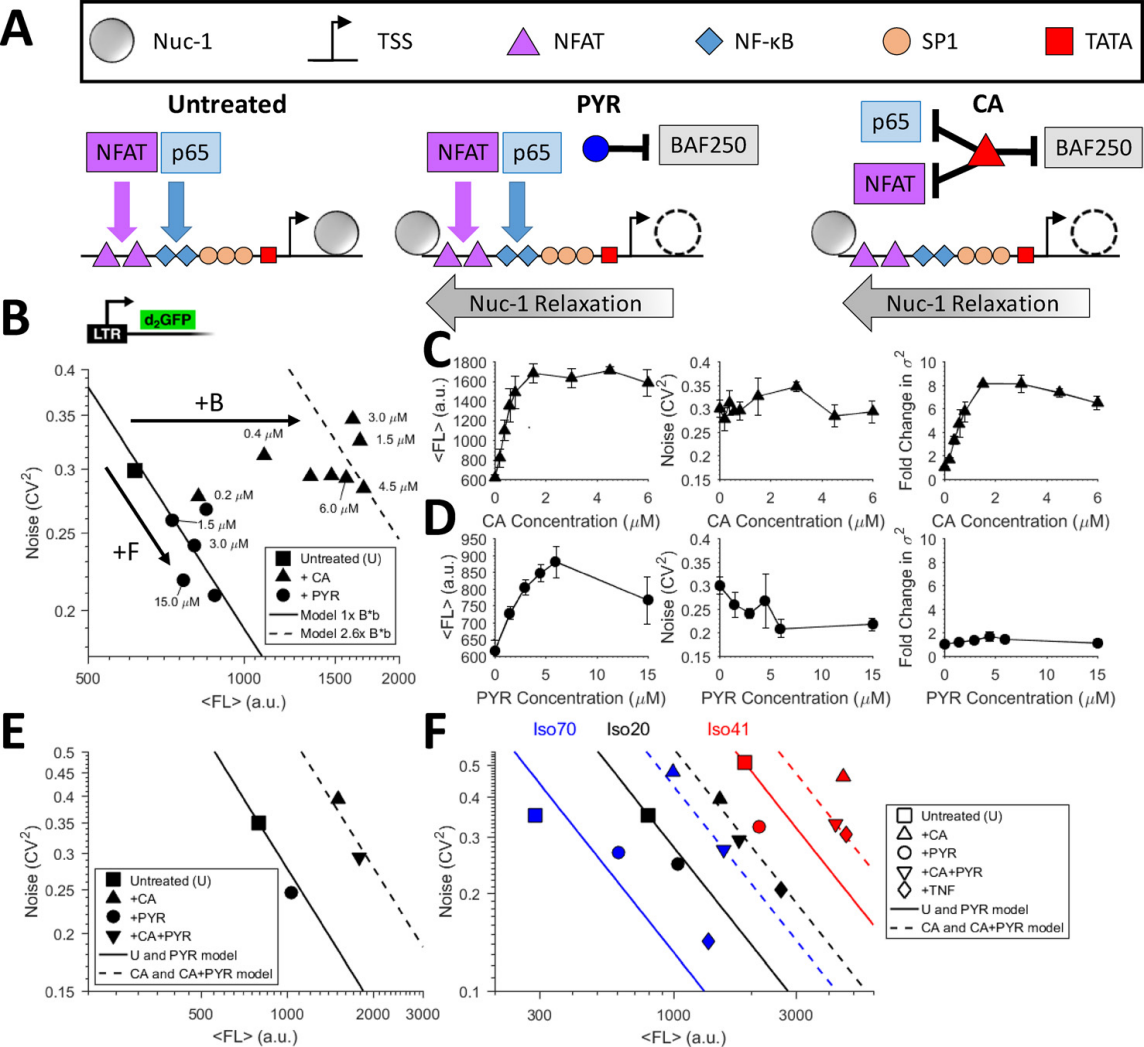
Pyrimethamine increases the LTR transcriptional burst frequency, while Caffeic acid phenethyl ester increases the transcriptional burst size. (a) Schematic of BAF inhibitor treatments on the HIV LTR promoter. Both Pyrimethamine (PYR) and Caffeic acid phenethyl ester (CA) degrade the BAF250a subunit, but CA is also known to inhibit activation through NF-κB and NFAT pathways. Inhibition of the BAF250a subunit relieves nucleosome occupancy upstream of the HIV LTR promoter and enables initiation of transcription. (b) Addition of increasing concentrations of PYR to a Ld2G isoclone follows a stable burst size model line with increasing frequency (fold increase = 1.44×) as noise decreases and d2GFP fluorescence increases. Addition of varying concentrations of CA to the same isoclone increases fluorescence with a constant noise level. (c) GFP fluorescence increase with CA treatment and constant noise is maintained by increasing variance up to 8× at concentrations above 1.5 *μ*M CA. (d) GFP fluorescence peaks at a concentration of about 5 *μ*M PYR, and the noise decreases with the increasing concentration. Variance remains constant with no increase with increasing concentrations of PYR. PYR follows a model of pure burst frequency modulation through BAF inhibition. (e) Combination treatment of CA+PYR displays an additive increase in both the burst size and the frequency, suggesting non-antagonistic drug activity. (f) Three isoclones of LTR-d2GFP (Iso 70, 20, and 41) show consistent shifts in noise with PYR, CA, and PYR+CA addition. The burst size and frequency change depending on differences in the clonal integration site on the high or low end of gene expression. All measurements were performed in duplicate with the mean and standard error plotted. The panel legend is applicable to the treatments of each of the three isoclones.

Although both PYR and CA target and degrade BAF250a, they are different in that CA has been reported to sequester nuclear factor-κB (NF-κB) to the cytoplasm and inhibit both the DNA-binding and transcriptional activity of nuclear factor of activated T-cells (NFAT) [Fig. 2(a)].^34,35^ Both NF-κB and NFAT are potent activators of the LTR and integral to transcriptional initiation of latent HIV.^36^ To measure gene expression noise with the two BAF inhibitor compounds, we performed dose dependent treatments of clonal populations of Jurkat T-cells integrated with the HIV LTR driving a destabilized d2GFP (LTR-d2GFP or Ld2G). Cells were treated with PYR and CA for 24 h, and noise was quantified using flow cytometry^14^ [Fig. 2(b), Methods section]. The BAFi compounds display distinct, continuous, and dose-dependent noise modulating trends: CA increases both mean fluorescence and variance in tandem while conserving a strictly constant noise level [Figs. 2(b) and 2(c)]. Lethality has been reported at high CA concentrations of more than 20–30 *μ*M,^37^ but cells exposed to concentrations of up to 6 *μ*M remained viable and display shifts in mean fluorescence (⟨FL⟩), which yield a ratio of B_CA_/B_Untreated_ = ∼2.6 using Eq. (1) with constant CV^2^ and F. In contrast, PYR follows a constant B model line [Eq. (2)], with a 1/⟨FL⟩ dependence expected for a pure increase in k_on_ or F by an activator [Figs. 2(b) and 2(d) and Eq. (2)].^23,26^ The dose response data result in a maximum of F_PYR_/F_Untreated_ = ∼1.44 using Eq. (2). The results suggest that although both drugs target degradation of BAF250a and regulate nucleosome occupancy in the LTR, PYR and CA provide orthogonal tuning of episodic transcriptional bursts—PYR tunes F, while CA tunes B [Fig. 2(b) and Eqs. (1) and (2)]. NF-κB and NFAT are among the major activating transcription factors binding κB binding sites on the LTR promoter.^38^ Inhibiting these initiation factors decreases transcriptional initiation, limits the increase in F with CA inhibition of BAF (as observed with PYR), and results in constant F with increasing B (compared to noise of the untreated promoter).

### CA and PYR are non-antagonistic and additively tune the burst size and frequency across multiple integration sites

To test the ability to tune both F and B simultaneously, we treated cells with combinations of PYR and CA in three different LTR-d2GFP isoclone populations named iso 70 (blue), iso 20 (black), and iso 41 (red) [Figs. 2(e) and 2(f)]. If treatments are non-antagonistic in their mechanisms for tuning noise, the combination of PYR and CA would independently tune F and B, respectively. PYR alone increases frequency, and despite common inhibition of BAF250a, an interaction with CA is undetected with noise shifts indicating additive B + F [Fig. 2(e)]. CA, PYR, and CA + PYR were added for 24 h to Ld2G iso 20, which was selected for its midrange expression level in a previously generated clonal library [Figs. 2(e) and 2(f)].^23,26^ For this clonal population, PYR consistently increased F with constant B. The combination of CA + PYR revealed an expected “slide” down the increased B model line following treatment with CA,^23,26,28^ suggesting that the control of the burst frequency and size by each compound is non-antagonistic and additive for simultaneous tuning of B + F in promoter noise [Fig. 2(e)]. Two additional isoclones on extreme ends of the fluorescence range in the clonal library were tested to show conservation of noise shifts with treatments across integration sites [Fig. 2(f)]. For CA treatment, B consistently increased with the highest shift in the lower abundance clone (Ld2G iso 70). Shifts for F and B + F were also fairly consistent across the isoclone expression range. Integration site differences suggest that the previously characterized integration site landscape for episodic transcription of the LTR constrains noise control in F-dominated (low abundance) and B-dominated (high abundance) regimes of the human genome.^23^ This suggests that the highly expressed iso 41 is already saturated in both F and B and cannot increase F further.^23,29^

### CA treatment demonstrates time-dependent regimes of noise tuning

**FIG. 3.**
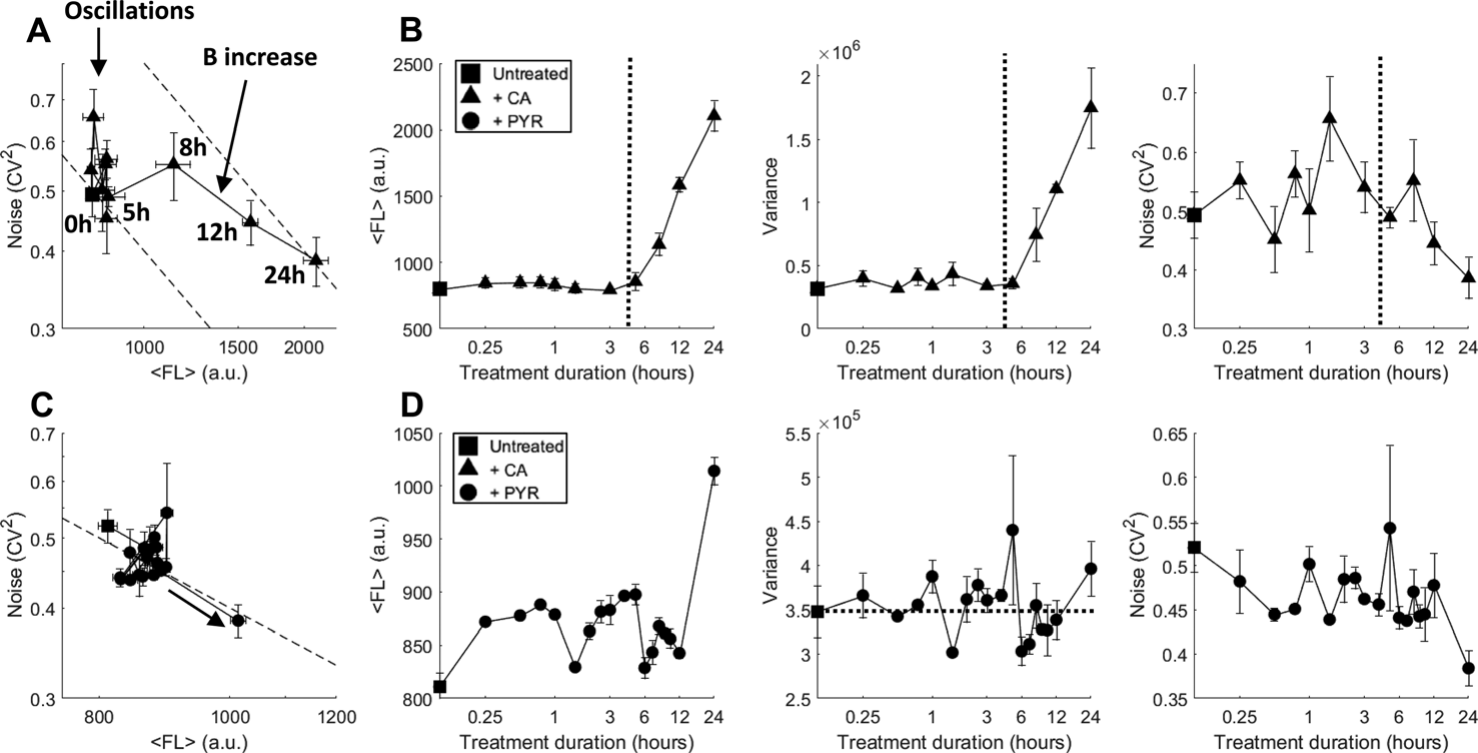
BAF inhibitor CA oscillates noise before increasing the burst size. (a) 24 h time-dependent CA treatment at different intervals reveals noise oscillations with a constant fluorescence level for treatment durations below 5 h. At 5–8 h, CA treatment increases the burst size while decreasing frequency before increasing frequency at a constant burst size at later treatment times. Dashed lines signify the constant burst size model line fits to the data at t = 0 and 24 h long duration treatment with the upper model using b*B × 2 compared to the lower model. (b) Mean fluorescence, GFP variance, and noise magnitude (CV^2^) as a function of CA treatment durations from panel (a). The vertical dashed line at 4 h separates between two time-dependent regimes of noise oscillations (left) and the increase in the burst size (right). [(c) and (d)] In contrast to CA, addition of PYR shows no oscillations for time-dependent treatments and increases burst frequency at longer 12–24 h treatment durations. All measurements were performed in duplicate with the mean and standard error plotted.

To examine whether noise tuning with PYR and CA treatments is monotonic with treatment duration times, we performed time-dependent measurements. Previous concentration-dependent tuning of noise resulted in steady state noise modulation after 24 h, yet it is unknown if CA and PYR treatment durations at a constant concentration result in continuous transient shifts in noise or if non-continuous tuning occurs at specific treatment durations. If dynamic modulation of BAF is continuous with time, time-dependent BAFi treatments would be equivalent to dose-dependent trends quantified for different concentrations (Fig. 2). Time-dependent treatments and measurements using flow cytometry were performed with 2 *μ*M CA, as 1–3 *μ*M was sufficient to saturate the increase in abundance and B observed in Fig. 2(b) [Figs. 3(a) and 3(b)]. Despite averaging three separate ensemble flow cytometry measurements, treatment durations for 0–5 h exhibited vertical oscillations of noise caused by oscillating variance at a constant mean abundance. The averaging of three independent and unsynchronized measurements dampened independent oscillation trends (Fig. S1). Extended treatment durations of 5–24 h show a steady increase in both abundance and variance [Figs. 3(b) and S1], where B increases with a decrease in F [Fig. 3(a)], ending with a decay of noise back to constant untreated (t = 0) noise levels along an increased and constant B model line (upper dashed line).

Besides its role of inhibiting the BAF complex, CA is also reported to potently inhibit NF-κB and NFAT in the cell.^34,35^ Marquez *et al.* have shown that CA inhibits NF-κB-dependent transcriptional activity and prevents NF-κB binding to DNA and transcriptional activity of a Gal4-p65 hybrid protein in treated Jurkat cells. In addition, CA inhibits both the DNA-binding and the transcriptional activity of NFAT.^35^ CA inhibition of NF-κB, NFAT, and BAF affects large resource pools required for genome-wide regulation and results in time-dependent active translocation of transcription factor from the cytosol to the cell nucleus^39,40^ and a modulation of the 2-state model before post-treatment steady states of global resources are achieved. Constant activation or inhibition of NF-κB has been shown to induce damped oscillations.^39,41^ Repeated time-dependent oscillations with CA treatments are shown in Fig. S1 and are observed to have different periodicity. As transient noise tuning is observed with ensemble cell measurements by flow cytometry, this suggests that a majority of the population is initially synchronized in its response to treatment.

In comparison to CA, time-dependent treatment with PYR over 24 h showed no significant changes in noise and mean fluorescence for the first 12 h [Figs. 3(c) and 3(d)]. An increase in mean abundance and F, consistent with dose-dependent frequency modulation observed in Fig. 2, is detected between 12 and 24 h of treatment, much later than the time-dependent tuning observed with CA [Fig. 3(b)].

### CA and PKC agonist combinations enhance the translational burst size

**FIG. 4.**
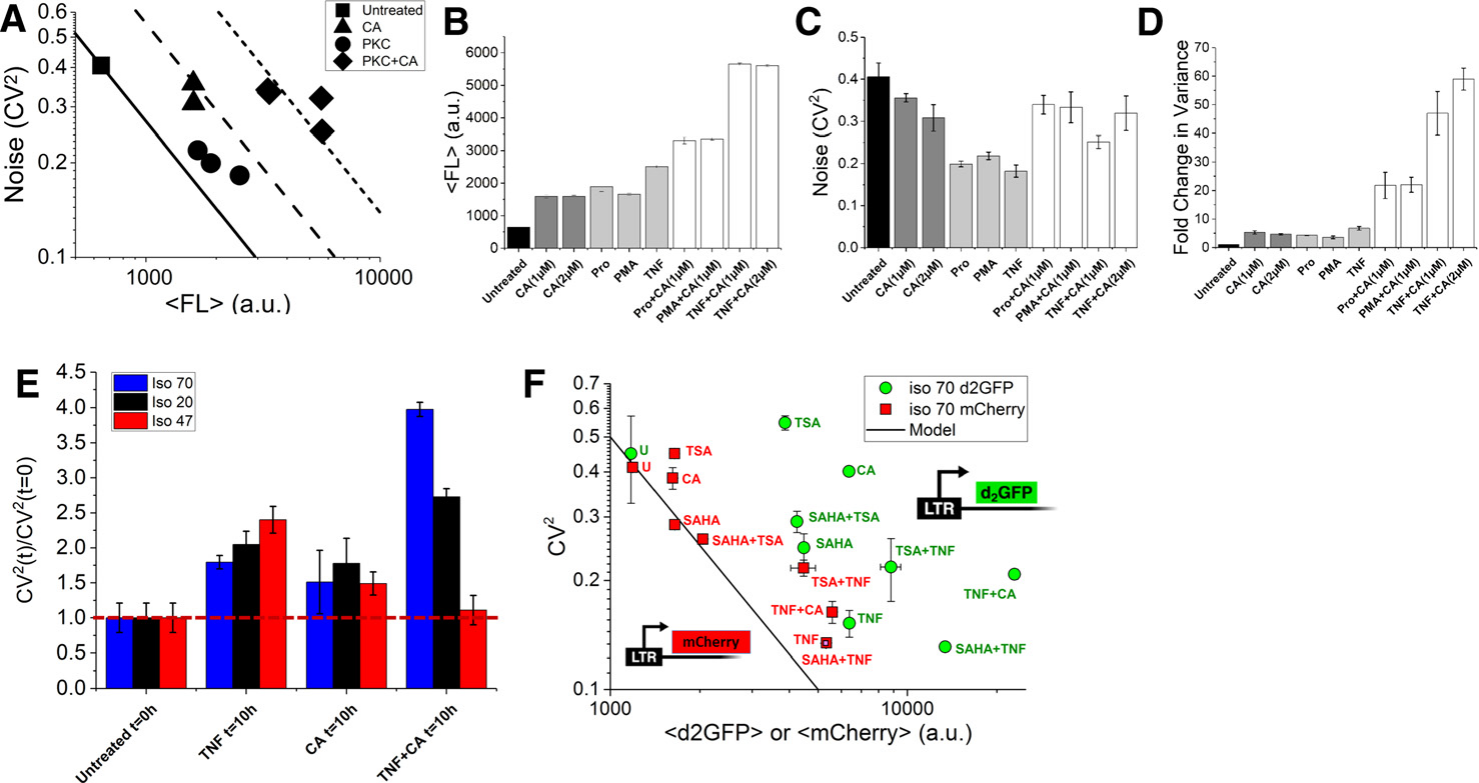
Effects of BAF inhibitor CA and PKC agonists on gene expression and noise (a) LTR-d2GFP isoclone 20 treated with CA (triangles) increases the burst size and mean protein abundance with constant noise. CA also inhibits transcriptional activation and burst frequency by NF-κB and NFAT, while PKC agonists (circles) increase both burst frequency and translational burst size. When used together, the increase in burst frequency by PKCa activation is negated by CA and accentuates the increase in the translational burst size. Experimental results using flow cytometry show that CA increases GFP fluorescence in combination with PKC agonists with constant noise to a larger total PKC+CA driven B*b model line (diamonds). From (a): (b) Iso 20 changes in fluorescence, (c) changes in the noise magnitude, and (d) Fold change in variance after the addition of various PKCa and CA drug combinations. Of the drug treatments tested, a maximal ∼63× increase in variance is observed with the addition of TNF with 2 *μ*M CA. (e) Transcriptional arrest with Flavopiridol to three LTR-d2GFP isoclone populations shows a shift in a ratio quantifying noise due to promoter fluctuations [CV^2^ (t)/CV^2^ (t = 0 h)]^47^ to higher amounts of post-transcriptional noise for both TNF and TNF+CA treatment. (f) Differential stability of two reporters, LTR-d2GFP and LTR-mCherry, allows the observation of CA treatment being strictly transcriptional with the model line deviation for d2GFP and a shift back to the model line for a stable mCherry reporter.^14,23^ On the other hand, consistent with panel (e) and transcriptional arrest measurements, TNF and CA+TNF shift back to the model line for stable mCherry reporter but still show post-transcriptional or translational noise by deviations from the model. For comparison, known chromatin modifiers TSA and SAHA and their combinations with TNF are measured to show their shifts with the mCherry measurement. All measurements were performed in duplicate with the mean and standard error plotted.

Protein kinase C (PKC) agonists TNF, Prostratin, and PMA potently initiate transcription by up-regulating production of NF-κB, AP-1, and NFAT.^42^ All three of these transcription factors have binding sites in the LTR promoter^42,43^ and result in transcriptional initiation and synergistic activation of HIV using drug cocktails.^14,15,21,44^ Transcriptional activators which purely increase F have been shown to move along constant B model lines (Figs. 1 and 2).^14,23,26,28^ Sole treatment of the clonal LTR population with transcriptional activators shows an increase in ⟨FL⟩ and F and a decrease in noise (CV^2^) [circles and dark grey bars, Figs. 4(a)–4(d)]. Upon combining treatment of CA and the PKC agonists (diamonds), CA pivots the noise coordinate of sole PKC treatments (circles) to a conserved constant noise level (dark grey and white bars), compared to CA treatment alone (triangles), with a large increase in both ⟨FL⟩ and variance [Figs. 4(a)–4(d)]. An 8× fold change in variance is observed for CA treatment alone compared to the untreated population [Figs. 2(b) and 4(d)] and in combination with TNF, Prostratin, or PMA reaches fold changes of ∼45–63× depending on the concentration of CA used [Fig. 4(d)].

PKC agonists increase phosphorylation of the elongation factor 1 (EF-1) family in the cytoplasm,^45^ resulting in increased translation rate^45,46^ and translational burst size (b). CA provides a dual role by BAFi relaxation of high nucleosome occupancy^18^ with simultaneous inhibition of transcriptional initiation. This allows for PKC agonists which typically increase both F and b to now shift to primarily increase translational bursts [Fig. 4(a)]. Here, the over-expression of NF-κB by PKC agonists is diverted away from strongly increasing F, while the increase in b by PKC agonists is more extenuated—presenting a third dial for tuning noise at both levels of transcription (B and F) and translation (b). Assuming that only b changes when TNF is combined with CA treatment, the measured ratio of b_TNF + CA_/b_CA_ = ∼3.25 and is comparable to the expected increase in the translation rate (k_p_) previously reported for PKC agonists^45,46^ [Eq. (1), assuming constant γ_m_].

To confirm that the combination of CA + PKC increases post-transcriptional noise, we utilized a reported method for distinguishing between alternate sources of noise of LTR promoter fluctuations versus birth/death of mRNA using time-dependent transcriptional arrest.^47^ Time-dependent noise measurements were performed under transcription arrest using Flavopiridol at a concentration of 10 *μ*M. For untreated clones harboring the LTR promoter driving d2GFP, this method previously revealed that the LTR is dominated by noise from promoter fluctuations and not mRNA birth/death or post-transcriptional processes.^47^ Three isoclones were tested for transcriptional arrest up for 10 h after pre-treatment with TNF, CA, and CA + TNF for 24 h [Fig. 4(e)]. Along with untreated controls, all three isoclones displayed noise dominated by promoter fluctuations with CA with a noise ratio approaching 1 (red dashed line). Conversely, TNF and CA + TNF showed increased noise ratios, suggesting a shift towards increased post-transcriptional noise sources away from a promoter fluctuation dominated picture.^47^ This result suggests that sole treatment with TNF simultaneously increases F and b, which is seen for the three TNF treated isoclones [Fig. 2(f)] and for each of the three PKC treatments [Fig. 4(a)]. The magnitude of the noise ratio shifts for TNF + CA [Fig. 4(e)] is inversely proportional to the untreated mean abundance and transcriptional burst size of the integration sites tested [Fig. 2(f)]. Isoclone 70 has the largest noise ratio, followed by iso 20. Iso 47 showed no shift as it is heavily dominated by saturated burst frequency and high transcriptional burst size at high abundances^23^ and thus remains dominated by promoter-fluctuations.

To confirm post-transcriptional noise modulation with CA + TNF treatment using an additional method, differential stability between two reporters driven by similar LTR promoters in the same clonal population was applied to distinguish between sources of noise.^14,23^ Here, while a destabilized d2GFP reporter captures short-lived processes, the stable LTR-mCherry expression can only report shifts in post-transcriptional noise. For iso 70, a variety of treatments including histone deacetylase (HDAC) inhibitors Trichostatin A (TSA) and Suberoylanilide Hydroxamic Acid (SAHA), TNF, and CA and their combinations show expected shifts of burst frequency and size with LTR-d2GFP [Fig. 4(f)]. Integrated in the same clonal population, noise from the stable LTR-mCherry reveals that treatments solely affecting transcription strictly adhere to the model line including HDAC inhibitors and CA, while combinations with TNF (CA + TNF, TSA + TNF, and SAHA + TNF) and TNF alone deviate from the model line suggesting post-transcriptional noise modulation^14^ [Figs. 4(f) and S2, consistent with Fig. 4(e)]. The results show that single treatment with CA and PKC agonists modifies LTR-d2GFP noise as previously shown (Figs. 2–4), but CA follows an ∼1/⟨FL⟩ model line for the stable LTR-mCherry [Fig. 4(f)]. The CA + PKC combination results in an increase in noise from the ∼1/⟨FL⟩ model in both the d2GFP and mCherry reporters, suggesting that post-transcriptional noise is being altered by the combination treatment.

**FIG. 5.**
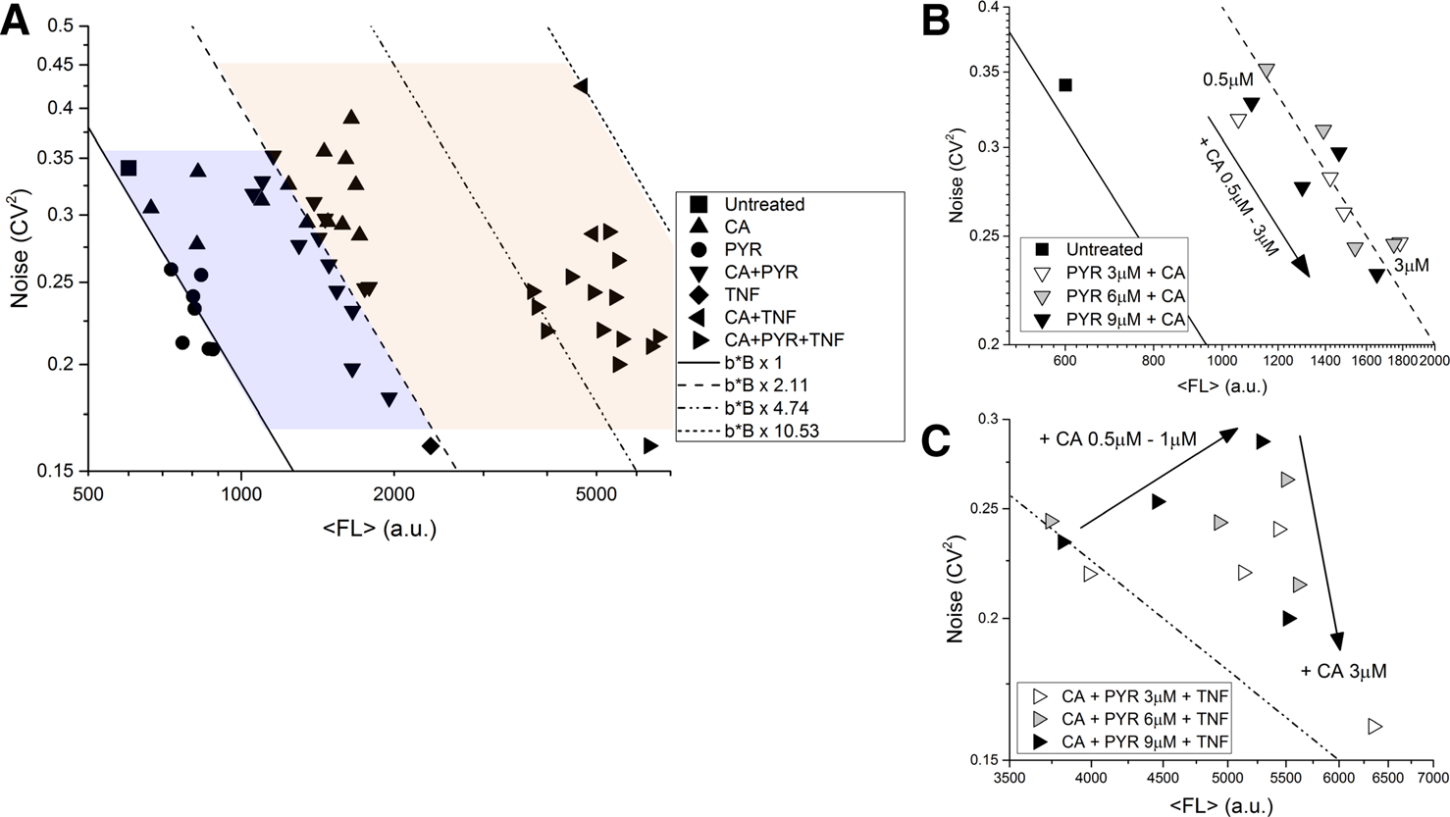
BAF inhibitors along with PKC agonists provide non-antagonistic tunable control in a transcriptional and translational noise phase space. (a) CA dose response (triangles) increases the transcriptional burst size and PYR (circles) moves along a fixed transcriptional burst model line. CA and PYR increase the size and frequency independently when combined in a drug treatment. The average standard error for duplicate measurements for CV^2^ is ±0.02 and for ⟨FL⟩ is ±111. Error bars are omitted to reduce clutter in the phase space. Lines represent 4 different constant burst size models. CA and PYR concentrations are identical to Fig. 2. CA+PYR uses PYR at 3, 6, and 9 *μ*M with CA from 0.5 to 3.0 *μ*M. TNF is used at a constant of 10 ng/mL, and CA+TNF is as used in Fig. 4. Finally, CA+PYR+TNF uses constant TNF with the CA+PYR combinations already described (right facing triangles). (b) Inset of PYR+CA dose response from (a). Shifts along a constant transcriptional burst size model line are seen for PYR combined with increasing CA. PYR alone moves along the fixed model line from the untreated cells. The increase in CA with constant PYR results in movement along the model line for different levels of PYR. Solid and dashed lines represent a constant burst size model from panel (a). (c) Inset of PYR+TNF+CA dose response from panel (a). The addition of increasing CA to constant concentrations of PYR+TNF results in an independent and extended movement of increased B followed by F in the far right portion of the noise space, requiring TNF treatment, for tuning B + b + F. Increasing CA concentration with constant PYR+TNF shows that movement to the right until maximal shift is achieved at 1–3 *μ*M CA at which point increased CA moves down similar to panel (b) (without TNF). All measurements were performed in duplicate. The dash-dotted line depicts a constant burst size model [also from panel (a)].

Finally, to demonstrate the ability to map out a detailed noise phase space by modulating F, B, and/or b, we combined PYR, CA, and TNF in different combinations and concentrations to test tunability and non-antagonistic modulation of LTR gene expression noise [Figs. 5(a)–5(c) and Methods section]. Different treatment combinations provide an extended tunable noise phase space, and notably, adding PYR to CA + TNF increases F in addition to the CA + TNF increase in B + b [right facing triangles, Fig. 5(a)] and is consistent with PYR acting alone [circles, Figs. 2 and 5(a)]. This further confirms that the two BAFis are non-antagonistic. Here, treatments expand the phase space to increase ⟨P⟩ up to 10× and CV^2^ decreases by half as compared to untreated values (Figs. 4 and 5). PYR + CA dose dependent treatments showed strict adherence to the increased B model line [downward facing triangles, Figs. 5(a) and 5(b)], and dose dependent treatment of CA added to PYR + TNF showed a shift of increasing B until ending by shifting downwards [Fig. 5(c)], consistent with the PYR + CA treatments [Fig. 5(b)]. Interestingly, increasing CA with either PYR or PYR + TNF results in movement down a constant B model line with increasing F [Figs. 5(b) and 5(c)]. The increases in B has already saturated with increasing CA [triangles, Figs. 2(b) and 5(a)], and non-antagonistic combinations with various PYR concentrations provide various amounts of increasing F (Figs. 2 and 5). This is seen by an immediate F increase when increasing CA concentrations are combined with different concentrations of PYR [Fig. 5(b)].

## DISCUSSION

This study builds upon observations that BAF inhibition remodels nucleosome occupancy of the HIV LTR promoter and activates transcription.^18,22^ With a recent noise drug screen showing that chromatin-modifying compounds enhance noise and transcriptional burst size,^14^ we assess the ability of nucleosome remodeling to precisely tune gene expression noise. BAFis demonstrated orthogonal modification of transcriptional noise with PYR increasing the transcriptional burst frequency and CA increasing the transcriptional burst size (Fig. 2). Both modulated noise in a dose response manner and their combination showed non-antagonistic and additive tuning of frequency and size. Time-dependent treatments with CA revealed a non-continuous, two-phase modulation of noise. At early treatment times, CA displayed noise oscillations at a constant mean fluorescence followed by an increase in the burst size at later times (Fig. 3). A large increase of expression variance up to an ∼63× fold change was observed for combinations of CA with TNF while conserving noise levels and provided burst size enhancement at both levels of transcription and translation (B + b, Fig. 4). Noise shifts were consistent between vastly different integration sites, suggesting that nucleosome occupancy targeting is applicable to a variety of genomic loci.

The ability of CA to inhibit transcriptional activation by simultaneously degrading the BAF250a subunit, remodeling nucleosome occupancy in the LTR promoter,^18^ and inhibiting transcriptional activators presents a new type of noise modulating compound that can redirect the noise modulating activity of another drug [e.g., TNF, Fig. 4(a)] and tune either transcriptional or translational noise when used in a noise drug cocktail.^14^ Simultaneous modulation of multiple noise sources of gene circuits advances the complexity and future applications for tuning noise. In a recent study, Wong *et al.* investigate noise from the LTR promoter with different activation levels from low basal expressing integration sites.^48^ TNF treatments show that both F and B can change depending on the integration site dependence and the local chromatin environment. They show that at low and high activatable integration sites, TNF treatment results in increased burst frequency and burst size, respectively,^23^ and that this is caused by differences in histone acetylation and buildup of primed and paused RNA Pol II. Taken with the findings in this study, the ability of TNF to increase both the transcriptional burst frequency and the size along with the translational burst size makes TNF (and potentially other PKC agonists) a versatile noise modulating candidate for stochastic design using distinct noise tuning strategies. These findings may contribute to the future engineering of stochasticity using multiple compounds while minimizing the number of input signals required.^5,6,49^

Exogenous BAFi compounds, including the FDA-approved PYR, provide the advantage of defined and finite treatment durations for tuning noise without the need to integrate synthetic gene vectors into a target cell population. For regulating decisions on finite timescales, such as stem cell differentiation and reactivation of latent HIV, limited windows for tuning noise may be advantageous. Furthermore, the oscillatory and transient behavior of noise with CA treatment demonstrates that engineering noise can be dynamic and may require temporal control for different applications.

Understanding the long-term implications of fine-tuning gene expression noise requires its extension and development into biological applications. As the LTR and other similar promoters, like the Cytomegalovirus (CMV),^43^ continue to be used in a variety of synthetic gene vectors, advanced methods for tuning noise in any gene of interest or regulatory motif may benefit from targeting BAF and nucleosome occupancy. Noise modulating drugs and BAFi cocktails have already been shown to synergize reactivation of HIV from latency.^14,22^ Additional applications that may benefit from tuning noise include the control of bacterial persistence,^50^ patterning during growth and development of multicellular tissues,^1^ reprogramming of stem cell pluripotency,^12^ and cancer gene therapies.^51^ With systems and synthetic biology advancing towards engineering epigenetics,^52^ this study highlights noise as a system-design element and provides principles for engineering stochasticity in biological systems.

## METHODS

### Cell lines and cell culture

Naïve Jurkats were obtained from ATCC, and LTR-d2GFP isoclone 20 was previously published in a noise drug screen^14^ and was kindly provided by the Weinberger Laboratory at the Gladstone Institute at UCSF along with isoclones 41, 47, and 70. Infection of naïve Jurkats for the production of LTR-d2GFP isoclones has been previously described.^23,26^ Both iso 41 and 47 are highly expressed integration sites, iso 70 is low, and iso 20 is mid-range.

### Growth condition of T-cells

Jurkat cells were grown in RPMI 1640 media supplemented with l-glutamine (Thermo Scientific), 10% fetal bovine serum (FBS), and 1% penicillin/streptomycin (Corning Cellgro). Cells were incubated with 5% CO_2_ at 37 °C.

### Drug treatments

The isoclone 20 cell line was used to test dose responses of Caffeic acid phenethyl ester (CA or CAPE) and Pyrimethamine (PYR) in Fig. 2. Both CA and PYR were acquired from Cayman Chemical. CA concentrations ranged from 0.1 *μ*M to 6 *μ*M, and PYR concentrations ranged from 1.5 *μ*M to 15 *μ*M. These concentrations were chosen based on the viability of the cells at certain drug concentrations. For treatments combining more than one drug, CA + PYR uses PYR at 3, 6, and 9 *μ*M with CA from 0.5 to 3.0 *μ*M. TNF (R&D Systems) was used at a constant of 10 ng/mL, and CA + TNF used in Fig. 4 uses CA at both 1 and 2 *μ*M. CA + PYR + TNF uses constant TNF with the CA + PYR combinations described. Prostratin was used at 3 *μ*M, and PMA used at 200 ng/mL, both acquired from Cayman Chemical. Cells were grown to a density of ∼1 × 10^6^ cells/mL before being transferred to 24 well plates for treatment. Measurements were performed on a BD LSRFortessa flow cytometer after 24 h of drug treatment.

Time-lapse experiments were also performed on isoclone 20 using PYR and CA. Flow cytometry was performed to measure the response from seventeen different time points for each drug. Cells were grown to a density of ∼1 × 10^6^ cells/mL before being transferred to 24 well plates for treatment. Drug was added at set time intervals, and measurements were all performed after 24 h.

All experiments included a Naïve Jurkat and untreated controls.

### Stochastic modeling and simulations

Using Eqs. (1) and (2), the relationship between CV^2^ and ⟨FL⟩ can be modelled as ${CV}^{2}=bB/\left\langle P\right\rangle$. With constant transcriptional and translational burst sizes, the model is a straight line with a slope of −1 on a log-log scale.

Gillespie algorithm was used to perform stochastic simulations.^53^ The simulation of the 2-state model of Fig. 1(a) starts with both steady state mRNA and protein levels and with the gene in the OFF state. It stops after 9 h of simulation time, and the last 6 h of data are analyzed to simulate a random starting condition within the biophysical range. Data resolution is one sample point per minute. Relevant parameters used for this system are listed in Table S1 and adapted from the study of Dar *et al.*^23^ For each noise modulating scenario simulated in Fig. 1, a total of 10 000 simulations were performed. The histograms of Fig. 1(b) take all simulated data points into consideration, while the scatter plots of Fig. 1(c) use the mean value of each population.

### Extrinsic noise filtering and autofluorescence correction

For flow cytometry data, FCS Express 5 was used to analyze a region of interest (ROI) containing the highest concentration of cells from each treatment sample. This ROI contains about 3000 cells of 50 000 total live cells collected per sample. The mean and variance of the fluorescence within the ROI are calculated from each treatment. Next, the values are corrected using the following formula:

$$\mu_{\text{corr}}=\mu_{\text{treat}}-\mu_{N},$$

$$\sigma_{\text{corr}}^{2}=\sigma_{\text{treat}}^{2}-\sigma_{N}^{2},$$

where $\mu_{\text{corr}}$ and $\sigma_{\text{corr}}^{2}$ stand for the corrected mean and variance for each treatment, $\mu_{\text{treat}}$ and $\sigma_{\text{treat}}^{2}$ stand for the raw mean and variance for each treatment, and $\mu_{N}$ and $\sigma_{N}^{2}$ stand for the mean and variance of a non-fluorescent naïve Jurkat sample. The sample mean fluorescence is $\mu_{\text{corr}}$, and the sample CV^2^ is calculated as $\sigma_{\text{corr}}^{2}/\mu_{\text{corr}}^{2}$. The correction has been previously defined in the supporting material in the study of Newman *et al.*^54^

### Confirmation of the post-transcriptional noise shift by CA + TNF

Confirmation of noise shifts from primarily affecting promoter fluctuations (CA, PYR, and TNF) to post-transcriptional sources was performed using two different noise modulation methods:

1. Transcriptional arrest^47^
  After 24 h of treatments, Flavopiridol was used in tandem to halt transcription prior to quantifying noise. Three Jurkat isoclones were used to demonstrate the effects of noise modulation at high (isoclone 47), low (isoclone 70), and moderate (isoclone 20) intensities of d2GFP fluorescence. The cells were treated for 24 h with final concentrations of TNF at 10 ng/mL, CA at 2 *μ*M/mL, and Flavopiridol at 10 *μ*M. Treatment with CA and TNF occurred 24 h before performing flow cytometry, and Flavopiridol was added 10 h before the measurement. Quantification and analysis of post-transcriptional noise were performed as previously reported.^47^
2. Differential stability two-reporter system^14^
  A previous report used two reporters, a destabilized d2GFP and a stable mCherry off of two identical LTR promoters in the same clonal cell population to identify which noise increased upon treatments.^14^ The stable mCherry filters promoter fluctuation noise and is dominated by post-transcriptional noise, specifically translational bursting. Compounds targeting transcription will change d2GFP noise with mCherry remaining constrained to an ∼1/⟨FL⟩ model line, while post-transcriptional noise modulators will increase noise in both d2GFP and mCherry channels.

Ethics approval was not required to perform this research.

## SUPPLEMENTARY MATERIAL

See supplementary material for additional supplementary figures and table.

## References

[1] H. M. Meyer and A. H. Roeder , Front. Plant Sci. 5, 420 (2014); 10.3389/fpls.2014.00420 25250034 PMC4157614

[2] G. Balazsi , A. van Oudenaarden , and J. J. Collins , Cell 144(6), 910 (2011); 10.1016/j.cell.2011.01.030 21414483 PMC3068611

[3] P. B. Gupta , C. M. Fillmore , G. Jiang , S. D. Shapira , K. Tao , C. Kuperwasser , and E. S. Lander , Cell 146(4), 633 (2011). 10.1016/j.cell.2011.07.026 21854987

[4] B. B. Kaufmann and A. van Oudenaarden , Curr. Opin. Genet. Dev. 17(2), 107 (2007). 10.1016/j.gde.2007.02.007 17317149

[5] K. F. Murphy , R. M. Adams , X. Wang , G. Balazsi , and J. J. Collins , Nucleic Acids Res. 38(8), 2712 (2010). 10.1093/nar/gkq091 20211838 PMC2860118

[6] T. Lu , M. Ferry , R. Weiss , and J. Hasty , Phys. Biol. 5(3), 036006 (2008). 10.1088/1478-3975/5/3/036006 18698117

[7] M. Acar , J. T. Mettetal , and A. van Oudenaarden , Nat. Genet. 40(4), 471 (2008). 10.1038/ng.110 18362885

[8] G. M. Suel , R. P. Kulkarni , J. Dworkin , J. Garcia-Ojalvo , and M. B. Elowitz , Science 315(5819), 1716 (2007). 10.1126/science.1137455 17379809

[9] N. Vardi , S. Levy , M. Assaf , M. Carmi , and N. Barkai , Curr. Biol. 23(20), 2051 (2013). 10.1016/j.cub.2013.08.043 24094854

[10] K. H. Kim , K. Choi , B. Bartley , and H. M. Sauro , IEEE Trans. Biomed. Circuits Syst. 9(4), 497 (2015). 10.1109/TBCAS.2015.2461135 26372647

[11] P. M. Caveney , S. E. Norred , C. W. Chin , J. B. Boreyko , B. S. Razooky , S. T. Retterer , C. P. Collier , and M. L. Simpson , ACS Synth. Biol. 6(2), 334–343 (2016). 10.1021/acssynbio.6b00189 27690390

[12] Z. S. Singer , J. Yong , J. Tischler , J. A. Hackett , A. Altinok , M. A. Surani , L. Cai , and M. B. Elowitz , Mol. Cell. 55(2), 319 (2014). 10.1016/j.molcel.2014.06.029 25038413 PMC4104113

[13] S. Wu , K. Li , Y. Li , T. Zhao , T. Li , Y. F. Yang , and W. Qian , PLoS Comput. Biol. 13(6), e1005585 (2017). 10.1371/journal.pcbi.1005585 28665997 PMC5513504

[14] R. D. Dar , N. N. Hosmane , M. R. Arkin , R. F. Siliciano , and L. S. Weinberger , Science 344(6190), 1392 (2014). 10.1126/science.1250220 24903562 PMC4122234

[15] D. Boehm , V. Calvanese , R. D. Dar , S. Xing , S. Schroeder , L. Martins , K. Aull , P. C. Li , V. Planelles , J. E. Bradner , M. M. Zhou , R. F. Siliciano , L. Weinberger , E. Verdin , and M. Ott , Cell Cycle 12(3), 452 (2013). 10.4161/cc.23309 23255218 PMC3587446

[16] I. Tirosh and N. Barkai , Genome Res. 18(7), 1084 (2008); 10.1101/gr.076059.108 18448704 PMC2493397

[17] L. Ho and G. R. Crabtree , Nature 463(7280), 474 (2010); 10.1038/nature08911 20110991 PMC3060774

[18] H. Rafati , M. Parra , S. Hakre , Y. Moshkin , E. Verdin , and T. Mahmoudi , Plos Biol. 9(11), e1001206 (2011). 10.1371/journal.pbio.1001206 22140357 PMC3226458

[19] U. Mbonye and J. Karn , Virology 454–455, 328 (2014). 10.1016/j.virol.2014.02.008 PMC4010583 24565118

[20] C. Kadoch and G. R. Crabtree , Sci. Adv. 1(5), e1500447 (2015); 10.1126/sciadv.1500447 26601204 PMC4640607

[21] C. A. Spina , J. Anderson , N. M. Archin , A. Bosque , J. Chan , M. Famiglietti , W. C. Greene , A. Kashuba , S. R. Lewin , D. M. Margolis , M. Mau , D. Ruelas , S. Saleh , K. Shirakawa , R. F. Siliciano , A. Singhania , P. C. Soto , V. H. Terry , E. Verdin , C. Woelk , S. Wooden , S. Xing , and V. Planelles , PLoS Pathogens 9(12), e1003834 (2013). 10.1371/journal.ppat.1003834 24385908 PMC3873446

[22] M. Stoszko , E. De Crignis , C. Rokx , M. M. Khalid , C. Lungu , R. J. Palstra , T. W. Kan , C. Boucher , A. Verbon , E. C. Dykhuizen , and T. Mahmoudi , EBioMedicine 3, 108 (2016). 10.1016/j.ebiom.2015.11.047 26870822 PMC4739437

[23] R. D. Dar , B. S. Razooky , A. Singh , T. V. Trimeloni , J. M. McCollum , C. D. Cox , M. L. Simpson , and L. S. Weinberger , Proc. Natl. Acad. Sci. U. S. A. 109(43), 17454 (2012). 10.1073/pnas.1213530109 23064634 PMC3491463

[24] L. S. Weinberger , J. C. Burnett , J. E. Toettcher , A. P. Arkin , and D. V. Schaffer , Cell 122(2), 169 (2005); 10.1016/j.cell.2005.06.006 16051143

[25] M. L. Simpson , C. D. Cox , and G. S. Sayler , J. Theor. Biol. 229(3), 383 (2004). 10.1016/j.jtbi.2004.04.017 15234205

[26] A. Singh , B. Razooky , C. D. Cox , M. L. Simpson , and L. S. Weinberger , Biophys. J. 98(8), L32 (2010). 10.1016/j.bpj.2010.03.001 20409455 PMC2856162

[27] R. Skupsky , J. C. Burnett , J. E. Foley , D. V. Schaffer , and A. P. Arkin , PLoS Comput. Biol. 6(9), e1000952 (2010); 10.1371/journal.pcbi.1000952 20941390 PMC2947985

[28] R. D. Dar , S. M. Shaffer , A. Singh , B. S. Razooky , M. L. Simpson , A. Raj , and L. S. Weinberger , PLoS One 11(7), e0158298 (2016). 10.1371/journal.pone.0158298 27467384 PMC4965078

[29] R. D. Dar , B. S. Razooky , L. S. Weinberger , C. D. Cox , and M. L. Simpson , PLoS One 10(10), e0140969 (2015). 10.1371/journal.pone.0140969 26488303 PMC4619080

[30] E. M. Ozbudak , M. Thattai , I. Kurtser , A. D. Grossman , and A. van Oudenaarden , Nat. Genet. 31(1), 69 (2002). 10.1038/ng869 11967532

[31] C. D. Cox , J. M. McCollum , M. S. Allen , R. D. Dar , and M. L. Simpson , Proc. Natl. Acad. Sci. U. S. A. 105(31), 10809 (2008). 10.1073/pnas.0804829105 18669661 PMC2504843

[32] E. C. Dykhuizen , L. C. Carmody , N. Tolliday , G. R. Crabtree , and M. A. Palmer , J. Biomol. Screen 17(9), 1221 (2012). 10.1177/1087057112455060 22853929 PMC4097195

[33] R. J. Conrad , P. Fozouni , S. Thomas , H. Sy , Q. Zhang , M. M. Zhou , and M. Ott , Mol. Cell 67(6), 1001–1012 (2017). 10.1016/j.molcel.2017.07.025 28844864 PMC5610089

[34] K. Natarajan , S. Singh , T. R. Burke, Jr. , D. Grunberger , and B. B. Aggarwal , Proc. Natl. Acad. Sci. U. S. A. 93(17), 9090 (1996). 10.1073/pnas.93.17.9090 8799159 PMC38600

[35] N. Marquez , R. Sancho , A. Macho , M. A. Calzado , B. L. Fiebich , and E. Munoz , J. Pharmacol. Exp. Ther. 308(3), 993 (2004). 10.1124/jpet.103.060673 14617683

[36] R. F. Siliciano and W. C. Greene , Cold Spring Harb. Perspect. Med. 1(1), a007096 (2011); 10.1101/cshperspect.a007096 22229121 PMC3234450

[37] J. Wu , C. Omene , J. Karkoszka , M. Bosland , J. Eckard , C. B. Klein , and K. Frenkel , Cancer Lett. 308(1), 43 (2011). 10.1016/j.canlet.2011.04.012 21570765 PMC3144783

[38] D. S. Ruelas and W. C. Greene , Cell 155(3), 519 (2013); 10.1016/j.cell.2013.09.044 24243012 PMC4361081

[39] A. Hoffmann , A. Levchenko , M. L. Scott , and D. Baltimore , Science 298(5596), 1241 (2002). 10.1126/science.1071914 12424381

[40] D. E. Nelson , A. E. Ihekwaba , M. Elliott , J. R. Johnson , C. A. Gibney , B. E. Foreman , G. Nelson , V. See , C. A. Horton , D. G. Spiller , S. W. Edwards , H. P. McDowell , J. F. Unitt , E. Sullivan , R. Grimley , N. Benson , D. Broomhead , D. B. Kell , and M. R. White , Science 306(5696), 704 (2004). 10.1126/science.1099962 15499023

[41] S. Nikolov , J. Vera , O. Rath , W. Kolch , and O. Wolkenhauer , IET Syst. Biol. 3(2), 59 (2009); 10.1049/iet-syb.2008.0105 19292561

[42] L. Colin and C. Van Lint , Retrovirology 6, 111 (2009). 10.1186/1742-4690-6-111 19961595 PMC2797771

[43] K. Bohn-Wippert , E. N. Tevonian , M. R. Megaridis , and R. D. Dar , Nat. Commun. 8, 15006 (2017). 10.1038/ncomms15006 28462923 PMC5418578

[44] J. K. Chan , D. Bhattacharyya , K. G. Lassen , D. Ruelas , and W. C. Greene , PLoS One 8(10), e77749 (2013). 10.1371/journal.pone.0077749 24204950 PMC3813743

[45] R. C. Venema , H. I. Peters , and J. A. Traugh , J. Biol. Chem. 266(19), 12574 (1991). 2061327

[46] H. I. Peters , Y. W. Chang , and J. A. Traugh , Eur. J. Biochem. 234(2), 550 (1995). 10.1111/j.1432-1033.1995.550_b.x 8536702

[47] A. Singh , B. S. Razooky , R. D. Dar , and L. S. Weinberger , Mol. Syst. Biol. 8, 607 (2012). 10.1038/msb.2012.38 22929617 PMC3435505

[48] V. C. Wong , V. L. Bass , M. E. Bullock , A. K. Chavali , R. E. C. Lee , W. Mothes , S. Gaudet , and K. Miller-Jensen , Cell Rep. 22(3), 585 (2018). 10.1016/j.celrep.2017.12.080 29346759 PMC5812697

[49] K. F. Murphy , G. Balazsi , and J. J. Collins , Proc. Natl. Acad. Sci. U. S. A. 104(31), 12726 (2007). 10.1073/pnas.0608451104 17652177 PMC1931564

[50] N. Q. Balaban , J. Merrin , R. Chait , L. Kowalik , and S. Leibler , Science 305(5690), 1622 (2004). 10.1126/science.1099390 15308767

[51] A. Brock , S. Krause , and D. E. Ingber , Nat. Rev. Cancer 15(8), 499 (2015). 10.1038/nrc3959 26156637

[52] P. I. Thakore , J. B. Black , I. B. Hilton , and C. A. Gersbach , Nat. Methods 13(2), 127 (2016); 10.1038/nmeth.3733 26820547 PMC4922638

[53] D. T. Gillespie , J. Phys. Chem. 81(25), 2340 (1977). 10.1021/j100540a008

[54] J. R. Newman , S. Ghaemmaghami , J. Ihmels , D. K. Breslow , M. Noble , J. L. DeRisi , and J. S. Weissman , Nature 441(7095), 840 (2006). 10.1038/nature04785 16699522

